# Real-World Effectiveness, Documented Adverse Events, and Treatment Adherence of Semaglutide and Liraglutide in Omani Patients: A Retrospective Observational Study

**DOI:** 10.3390/jcm15207797

**Published:** 2026-10-09

**Authors:** Najwa Al-Himali, Hanan Al-Habsi, Ahmed Al Mushrafi, Amal Al Shidi, Ayat Abbas, Amjaad Al Kindi, Hamza Syed, Iman Mohammadi, Amna Al Subhi, Maisa Hamed Al Kiyumi

**Affiliations:** 1 Department of Pharmacy, Sultan Qaboos University Hospital, University Medical City, Muscat 123, Oman; n.alhimali@squ.edu.om; 2 Department of Pharmacy, Sultan Qaboos University, Muscat 123, Oman; 3 College of Medicine and Health Sciences, Sultan Qaboos University, Muscat 123, Oman; s131496@student.squ.edu.om (A.A.M.); s137962@student.squ.edu.om (A.A.K.); s142958@student.squ.edu.om (H.S.); s136788@student.squ.edu.om (A.A.S.); 4 Department of Primary Health Care, Ministry of Health, Muscat 100, Oman; r22161@resident.omsb.org (A.A.S.); r22160@resident.omsb.org (A.A.); emi.moh@gmail.com (I.M.); 5 Department of Family Medicine and Public Health, Sultan Qaboos University Hospital, University Medical City, Sultan Qaboos University, Muscat 123, Oman; 6 Department of Family Medicine and Public Health, College of Medicine and Health Sciences, Sultan Qaboos University, Muscat 123, Oman

**Keywords:** semaglutide, liraglutide, glucagon-like peptide-1 receptor agonist, obesity, type 2 diabetes mellitus, treatment adherence, treatment persistence, propensity score, overlap weighting, retrospective cohort study

## Abstract

**Background**: Glucagon-like peptide-1 receptor agonists (GLP-1 RAs) are widely used in obesity and type 2 diabetes mellitus (T2DM), but comparative real-world evidence from the Middle East remains limited. We compared the effectiveness, documented adverse events, adherence and persistence of once-weekly semaglutide and once-daily liraglutide in Omani adults. **Methods**: Retrospective cohort study of adults treated with semaglutide or liraglutide at Sultan Qaboos University Hospital between January 2024 and December 2025. The primary outcome was change in body mass index (BMI) at 3 and 6 months; change in body weight, percentage total body weight loss and the proportions achieving at least 5% and at least 10% weight loss. Because treatment indication and exposure differed markedly between groups, the primary comparison used propensity-score overlap weighting with additional covariate adjustment, implemented in a design-based general linear model. A mixed model for repeated measures supported the primary analysis and handled missing visits under a missing-at-random assumption, with a delta-adjusted pattern-mixture analysis used to test departures from that assumption. Persistence was analysed with Kaplan–Meier estimation and Cox regression adjusted for baseline covariates only. Secondary outcomes were corrected for multiplicity using the Benjamini–Hochberg procedure. Analyses are labelled throughout as primary, prespecified secondary, or post hoc exploratory. No patient received semaglutide 2.4 mg weekly, the dose licenced for chronic weight management, due to unavailability during the study period. **Results**: Of 287 treatment records supplied, 257 unique patients were analysed (liraglutide *n* = 135; semaglutide *n* = 122). Semaglutide was associated with a greater reduction in BMI than liraglutide at 3 months (adjusted difference −0.60 kg/m^2^, 95% CI −1.12 to −0.07; *p* = 0.026) and at 6 months (−1.42 kg/m^2^, 95% CI −2.60 to −0.24; *p* = 0.019). In clinical units, the adjusted difference in body weight was −1.70 kg at 3 months (95% CI −3.10 to −0.29; *p* = 0.018) and −3.66 kg at 6 months (95% CI −6.72 to −0.60; *p* = 0.020). At 6 months, 56.5% of semaglutide-treated and 24.7% of liraglutide-treated patients had lost at least 5% of body weight (*p* < 0.001), and 30.4% versus 4.9% had lost at least 10% (*p* < 0.001). The mixed model gave the same conclusion (group-by-time interaction *p* < 0.001). No statistically detectable difference in glycated hemoglobin HbA1c change was observed at either time point (*p* = 0.132 and *p* = 0.158; interaction *p* = 0.360). Of eight secondary cardiometabolic outcomes, only total cholesterol at 3 months remained significant after correction for multiplicity (adjusted *p* = 0.024). Documented adverse events were infrequent and did not differ between groups (12.6% versus 8.2%, *p* = 0.251). Hypoglycaemia occurred in seven liraglutide-treated and one semaglutide-treated patient (*p* = 0.069 by exact test) and was not significant after weighting and adjustment for background insulin or sulfonylurea use (*p* = 0.345). Crude discontinuation was 48.9% with liraglutide versus 9.0% with semaglutide, but median exposure was 17 versus 6 months. After accounting for time at risk, no difference in persistence was statistically apparent (log-rank *p* = 0.328; adjusted hazard ratio for semaglutide 1.60, 95% CI 0.61 to 4.19). Documented adherence was higher with semaglutide (95.9% versus 83.7%, *p* = 0.001). **Conclusions**: In this cohort, semaglutide was associated with a modestly greater reduction in BMI and body weight than liraglutide, and with a higher proportion of patients achieving clinically meaningful weight loss. Glycaemic and most cardiometabolic comparisons were inconclusive once confounding, multiplicity and limited precision were accounted for, and the large apparent difference in discontinuation was no longer statistically apparent after allowing for unequal time at risk. These findings are associative. They describe the drugs as prescribed in one health-care setting, at doses that are not pharmacologically equivalent, and should not be read as evidence of comparative causal superiority.

## 1. Introduction

Type 2 diabetes mellitus (T2DM) and obesity are increasingly recognised as overlapping diseases that share common pathophysiological and behavioural determinants. Globally, obesity is the principal driver of T2DM [1,2]. That relationship argues for therapeutic strategies that address both conditions at once, rather than separately. Glucagon-like peptide-1 receptor agonists (GLP-1 RAs) are one such class [3,4]. They mimic the endogenous GLP-1 hormone by stimulating insulin secretion, inhibiting glucagon release, delaying gastric emptying and increasing satiety, which gives a combined benefit in glycaemic control, weight loss and cardiovascular risk [1,5]. The two most commonly used agents in this class are liraglutide and semaglutide. Liraglutide is given by daily subcutaneous injection at doses up to 3.0 mg for weight management [6,7]. Semaglutide is a modified GLP-1 RA with 94% homology to native GLP-1; its higher albumin affinity and resistance to degradation by dipeptidyl peptidase-4 allow once-weekly subcutaneous dosing at doses up to 2.4 mg [8,9,10].

The two agents have been compared head-to-head in several trials and reviews [11,12,13,14]. Semaglutide Treatment Effect in People with Obesity STEP 8 was a randomised, open-label, 68-week, phase IIIb trial in 338 adults with overweight (BMI ≥ 27 kg/m^2^ with at least one weight-related comorbidity) or obesity (BMI ≥ 30 kg/m^2^) without diabetes. Once-weekly semaglutide 2.4 mg reduced baseline body weight by 15.8% compared with 6.4% for once-daily liraglutide 3.0 mg (estimated treatment difference −9.4%; 95% CI −12.0 to −6.8; *p* < 0.001), corresponding to mean weight losses of 15.3 kg and 6.8 kg from baseline weights of 102.5 kg and 103.7 kg. Semaglutide also produced larger reductions in HbA1c, waist circumference, fasting plasma glucose, plasma lipids and C-reactive protein. Adverse gastrointestinal events occurred in 84.1% of the semaglutide group and 82.7% of the liraglutide group. Most were mild to moderate, and discontinuation due to these events was lower with semaglutide (3.2% versus 12.6%) [15]. Adherence is itself a determinant of drug effectiveness. The STAY study, a large United States claims analysis, found that persistence with once-weekly injectable GLP-1 RAs was higher than with daily agents (*p* < 0.01), and that adherence at 6 and 12 months was also higher (51% versus 40% and 43% versus 31%) [16,17]. A meta-analysis of injectable GLP-1 RAs concluded that weekly administration should be preferred where feasible, because persistence is associated with a greater likelihood of reaching glycaemic targets [18]. More recent claims data have tempered this picture: among commercially insured adults without diabetes, overall persistence was 46.3% at 180 days and 32.3% at one year, with one-year persistence of 47.1% for semaglutide and 19.2% for liraglutide [19]. The international evidence is strong, but regional data from the Middle East remain sparse. A retrospective study from a quaternary centre in the Gulf found larger HbA1c reductions with semaglutide than liraglutide in routine practice, although the difference in weight reduction between arms was not statistically significant [2]. A Saudi single-centre cost-consequence analysis in obese prediabetic and diabetic patients reported greater weight loss and HbA1c reduction with semaglutide [20]. No study within Oman has compared semaglutide with liraglutide in patients with or without T2DM.

This study was designed to address that gap and to compare once-weekly subcutaneous semaglutide with once-daily subcutaneous liraglutide in Omani adults with respect to BMI, HbA1c, cardiometabolic parameters, adverse events, adherence and treatment persistence.

## 2. Materials and Methods

### 2.1. Study Design and Population

This was a single-centre retrospective observational cohort study at Sultan Qaboos University Hospital, a tertiary hospital in Muscat, Oman. Data were extracted over three months (April to June 2026) from the hospital’s electronic medical record.

Adults aged 18 years or over who were prescribed either semaglutide or liraglutide between January 2024 and December 2025 were eligible. Treatment was prescribed either for weight management or for glycaemic control, and both indications were eligible. No minimum baseline BMI was applied. Patients were excluded if they had a history of pancreatitis, a documented contraindication to GLP-1 RA therapy, or were pregnant or breastfeeding. Patients who discontinued early were retained in the cohort, so that persistence could be estimated without survivor bias.

**Figure 1:**
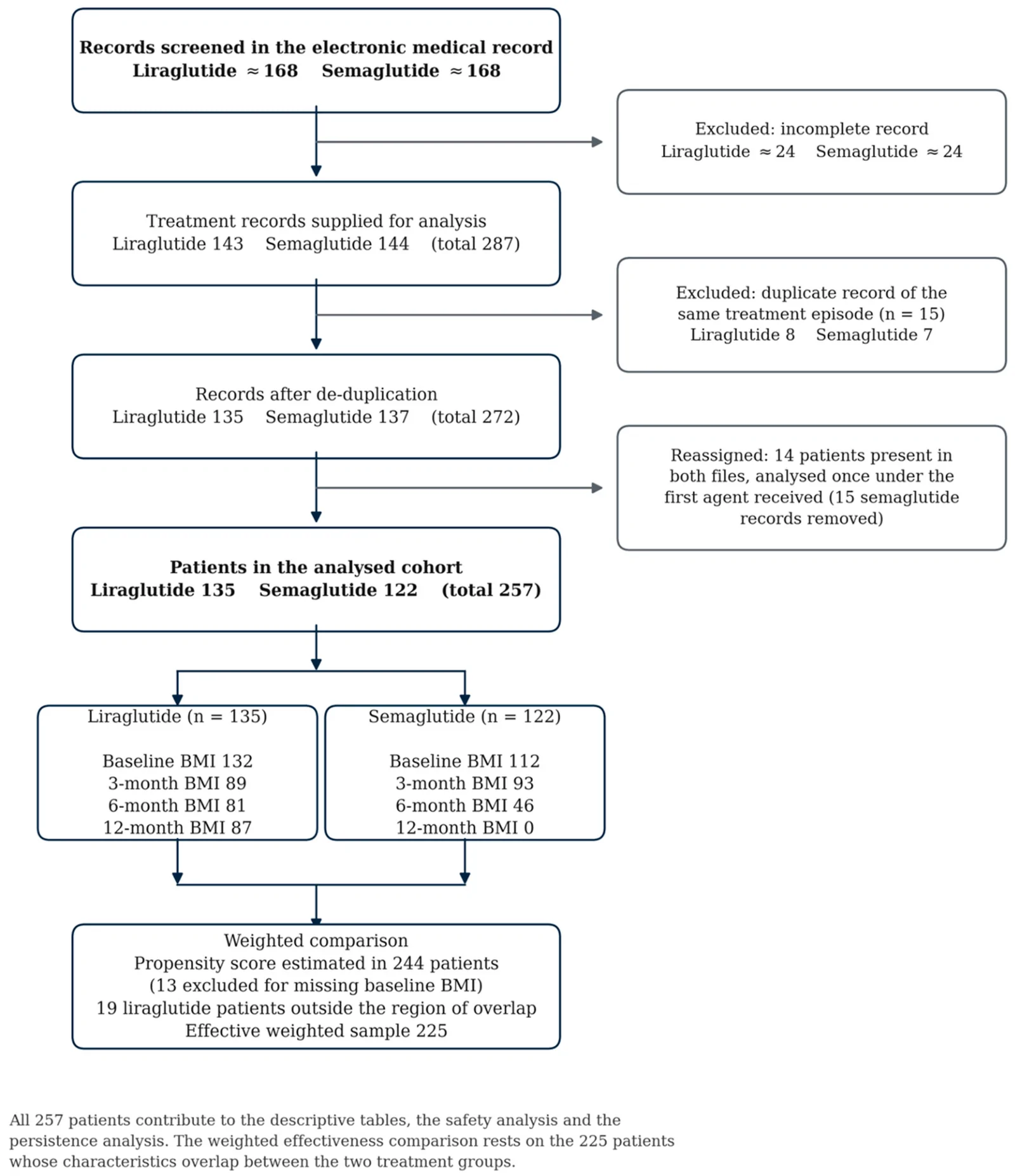
Participant flow through screening, de-duplication and analysis.

Several patients had more than one record in the source data. Records sharing a medical record number and age were treated as duplicate entries of the same episode, and the more complete record was kept. Fourteen patients appeared in both treatment files, having started liraglutide and later switched to semaglutide after a treatment-free interval of approximately one year. Each of these patients was analysed once, under the first GLP-1 RA received, together with the baseline and follow-up measurements belonging to that treatment period. Prior GLP-1 RA exposure was therefore not concurrent therapy and was not counted as a concomitant medication. Patient flow is shown in Figure 1.

All extracted data were anonymised before analysis. The study was conducted in accordance with the Declaration of Helsinki, and reporting follows the STROBE recommendations for observational studies [21].

### 2.2. Data Collection and Study Outcomes

Demographic characteristics, baseline clinical characteristics, treatment details, laboratory results and clinical outcomes were extracted from the TrakCare electronic medical record. Baseline measurements were those recorded before treatment initiation. Follow-up clinical and laboratory data were taken at approximately 3 and 6 months after initiation, where available, and at 12 months for liraglutide.

The primary outcome was change in BMI from baseline at 3 and 6 months. Secondary outcomes were change in HbA1c, triglycerides, total cholesterol, systolic and diastolic blood pressure, heart rate and estimated glomerular filtration rate (eGFR); the occurrence and type of adverse events; treatment adherence; and treatment persistence. BMI change was defined as the follow-up value minus the baseline value, so that a negative value denotes a reduction.

Because BMI alone is difficult to interpret clinically, change in body weight in kilograms and percentage total body weight loss were also prespecified, together with the proportions of patients achieving at least 5% and at least 10% loss of baseline body weight. These thresholds were chosen because losses of this magnitude are associated with measurable improvement in cardiovascular risk factors [22].

### 2.3. Definitions of Adherence, Persistence and Discontinuation

The three terms are used here as defined by Cramer and colleagues and are not interchangeable [23]. Adherence refers to the extent to which a patient takes the drug as prescribed. In this study, it was a binary variable recorded by the treating clinician at follow-up, based on the patient’s report of missed or skipped doses documented in the consultation note. Pharmacy dispensing and refill data were not available, so this measure reflects documented clinical impression rather than a calculated proportion of days covered, and it should be interpreted with that limitation in mind. Persistence refers to the time from initiation to discontinuation and was analysed as a time-to-event outcome. Discontinuation was defined as a documented decision to stop the GLP-1 RA; patients still receiving treatment at the time of data extraction were censored at that point.

### 2.4. Statistical Analysis

Analyses were performed in IBM SPSS Statistics version 32 (IBM Corp., Armonk, NY, USA). Distributions were assessed graphically and with the Shapiro–Wilk test. Most continuous variables departed from normality, so continuous data are summarised as median with interquartile range and compared with the Mann–Whitney U test, with age additionally reported as mean with standard deviation. Categorical data are summarised as *n* (%) and compared with the Pearson chi-square test, or the Fisher exact test where any expected cell count was below five. The number of patients contributing data is given for every variable and every time point, because these denominators vary.

Balance was assessed by standardised mean differences before and after weighting. A value below 0.10 was taken as balance. We note explicitly that a standardised mean difference of zero after overlap weighting is an algebraic property of the method rather than independent evidence that confounding has been removed [24,25]. Positivity was therefore examined directly: the distribution of the propensity score in each group is presented graphically, together with the distribution of the overlap weights and the effective sample size in each arm, calculated by the Kish formula as the square of the sum of the weights divided by the sum of their squares [26].

Weighted outcome models were fitted using the SPSS Complex Samples general linear model version 32, which treats the weights as sampling weights and returns design-based standard errors. The primary model for BMI change additionally included the same covariates used in the propensity model, giving a doubly robust estimate that remains valid if either the propensity model or the outcome model is correctly specified. Results are reported as the adjusted between-group difference in change with a 95% confidence interval. Inverse probability weighting, trimmed at the first and ninety-ninth percentiles, was run as a sensitivity analysis, as were an unweighted covariate-adjusted model and a generalised estimating equation with robust standard errors.

Responder outcomes were analysed by the chi-square or Fisher exact test and by overlap-weighted logistic regression. The two models for 10% weight loss were reduced to treatment group and baseline BMI, because only 9 and 18 events were available at 3 and 6 months and a model carrying ten covariates on that number of events would not be stable.

Because a mixed model cannot address missingness that depends on the unobserved outcome itself, a delta-adjusted pattern-mixture analysis was added to test how far the primary BMI result depends on the missing-at-random assumption [27]. Missing values of BMI change were replaced by the observed mean of the patient’s own treatment group, and a penalty (delta) was then added to the imputed values in the semaglutide group only, so that non-completers in that arm were assumed to have done progressively worse than completers. Delta was increased in steps until the between-group difference was no longer statistically significant; that value is reported as the tipping point. Multiple imputation was not performed separately, because the mixed model uses all observed visits under the same assumption and the pattern-mixture analysis addresses departure from it directly.

Treatment persistence was analysed by Kaplan–Meier estimation with the log-rank test, and by Cox proportional hazards regression. The primary persistence model was adjusted for baseline covariates only: age, sex, baseline BMI, diabetes, indication and background insulin or sulfonylurea use. Documented adherence was deliberately excluded because it is recorded after treatment begins and may lie on the causal pathway between drug choice and discontinuation, so adjusting for it risks overadjustment or collider bias. A model additionally adjusted for adherence is reported as a secondary, exploratory analysis. The proportional hazards assumption was tested with a time-dependent covariate. Because follow-up differed markedly between groups, discontinuation was also compared at a fixed horizon of 6 months, at which point both groups had comparable observation time. Fourteen patients received both drugs sequentially and were analysed once, under the first agent received. Because this discards potentially informative within-person data, the primary analysis was repeated after excluding these patients, with the propensity model refitted inside the reduced sample so that the weights belong to the sample in which they are used.

The primary outcome was prespecified as BMI change and was not subjected to multiplicity correction. The eight secondary cardiometabolic outcomes were corrected using the Benjamini–Hochberg false discovery rate procedure, and both raw and adjusted *p*-values are reported [28]. Model assumptions were checked by inspection of residuals, variance inflation factors for multicollinearity, and Cook’s distance for influential observations, with the primary model refitted after excluding cases whose Cook distance exceeded 4/*n*. All tests were two-sided with a significance threshold of 0.05.

### 2.5. Sample Size, Precision and Analysis Hierarchy

The sample comprised every eligible patient identified in the hospital record system during the study period, so achievable precision rather than a target sample size determined what the study could detect. With 89 and 92 patients contributing a 3-month BMI and an observed standard deviation of change of 1.63 kg/m^2^, the half-width of the 95% confidence interval for the primary comparison was 0.54 kg/m^2^ and the minimum difference detectable with 80% power at a two-sided alpha of 0.05 was 0.68 kg/m^2^. At 6 months, with 81 and 46 patients and a standard deviation of 2.51 kg/m^2^, the corresponding values were 1.22 and 1.30 kg/m^2^. The study was therefore adequately powered for the primary endpoint and clearly underpowered for the secondary, glycaemic and adverse-event comparisons.

To keep confirmatory and exploratory findings separate, analyses are labelled throughout as follows. The primary analysis is the overlap-weighted, covariate-adjusted between-group difference in BMI change at 3 and 6 months, together with the corresponding differences in body weight, percentage total body weight loss and responder proportions. Prespecified secondary analyses are HbA1c, the seven other cardiometabolic outcomes, documented adverse events, adherence and persistence. Post hoc sensitivity analyses are the pattern-mixture tipping point, exclusion of the 14 switchers, inverse probability weighting, the unweighted adjusted model, the generalised estimating equation and exclusion of influential observations. Exploratory analyses are the liraglutide-only 12-month trajectory and the modal-dose subgroup comparison, the latter presented as Supplementary Table S1.

## 3. Results

### 3.1. Patient Flow and Analysed Cohort

Approximately 168 records were screened in each treatment group, of which about 24 per group were incomplete and were removed, leaving 143 liraglutide and 144 semaglutide records. Fifteen records were duplicate entries of the same treatment episode and were removed, and a further 15 records belonging to the 14 patients who had received both drugs were removed so that each patient was counted once under the first agent received. The analysed cohort comprised 257 patients: 135 who received liraglutide and 122 who received semaglutide (Figure 1).

Baseline BMI could not be calculated for 13 patients because height was not recorded, so the propensity model was fitted in 244 patients. A further 19 liraglutide-treated patients had an estimated probability of receiving semaglutide of essentially zero and therefore received no weight; all 19 were patients whose background glucose-lowering therapy had not been documented by name. The weighted comparison therefore rests on 225 patients whose characteristics overlap between the two groups. All 257 patients contribute to the descriptive tables, the safety analysis and the persistence analysis.

### 3.2. Baseline Characteristics

Baseline characteristics are shown in Table 1. Patients receiving liraglutide had a mean age of 46.9 (SD 14.1) years and 60.7% were women; those receiving semaglutide had a mean age of 50.2 (SD 14.4) years and 64.8% were women. Median baseline BMI was 35.4 kg/m^2^ in the liraglutide group and 36.2 kg/m^2^ in the semaglutide group, and median baseline HbA1c was 7.9% and 7.8%. Diabetes was present in roughly three-quarters of each group.

Several characteristics differed. Hypertension, dyslipidaemia and non-alcoholic fatty liver disease were more common in the semaglutide group; cardiovascular disease was more common in the liraglutide group; and baseline total cholesterol was higher in the semaglutide group.

### 3.3. Treatment Characteristics and Concomitant Therapy

Treatment characteristics are shown in Table 2. The two groups differed sharply in why treatment was started and for how long it continued. Weight management was the indication in 55.6% of liraglutide-treated patients and 87.7% of those receiving semaglutide. Median treatment duration was 17 months with liraglutide and 6 months with semaglutide, and the median number of follow-up visits was 3 and 1. These differences are the main reason that unadjusted comparisons in this cohort cannot be interpreted as treatment effects.

The modal maintenance dose was 1.8 mg daily for liraglutide and 1.0 mg weekly for semaglutide. No patient reached the 2.4 mg weekly dose of semaglutide licenced for chronic weight management, because that presentation was not available in Oman during the study period. Background glucose-lowering therapy was documented by drug name for 87 liraglutide-treated and 90 semaglutide-treated patients; 21 liraglutide records indicated that other therapy was being taken but did not name it, so drug-class variables are reported as missing for those patients rather than as absent. Among patients with named therapy, insulin and sulfonylurea use did not differ significantly between groups.

### 3.4. Availability of Follow-Up Data

The number of patients contributing each outcome at each visit is given in Table 3. Attrition was substantial and unequal. At 6 months, BMI was available for 60.0% of the liraglutide group and 37.7% of the semaglutide group, and HbA1c for 53.3% and 14.8%. The semaglutide group had essentially no 12-month data because semaglutide became available later in the study period, so between-group comparison is restricted to 3 and 6 months.

Availability of follow-up data was related to baseline characteristics. Patients receiving semaglutide were more likely to have a 3-month BMI recorded (adjusted odds ratio 2.63, 95% CI 1.22 to 5.65; *p* = 0.013), while availability at 6 months tended to fall with older age (*p* = 0.057) and higher baseline BMI (*p* = 0.094). Missingness is therefore not completely at random, which is why the mixed model rather than complete-case analysis was used for the longitudinal comparisons.

### 3.5. Covariate Balance After Weighting

Before weighting, seven of the ten covariates in the propensity model had a standardised mean difference above the conventional threshold of 0.10, with treatment indication the most imbalanced at 0.788. After overlap weighting, every covariate was balanced exactly (Table 4). This is a property of overlap weights rather than a chance finding, and it is the reason they were chosen here.

**Figure 2:**
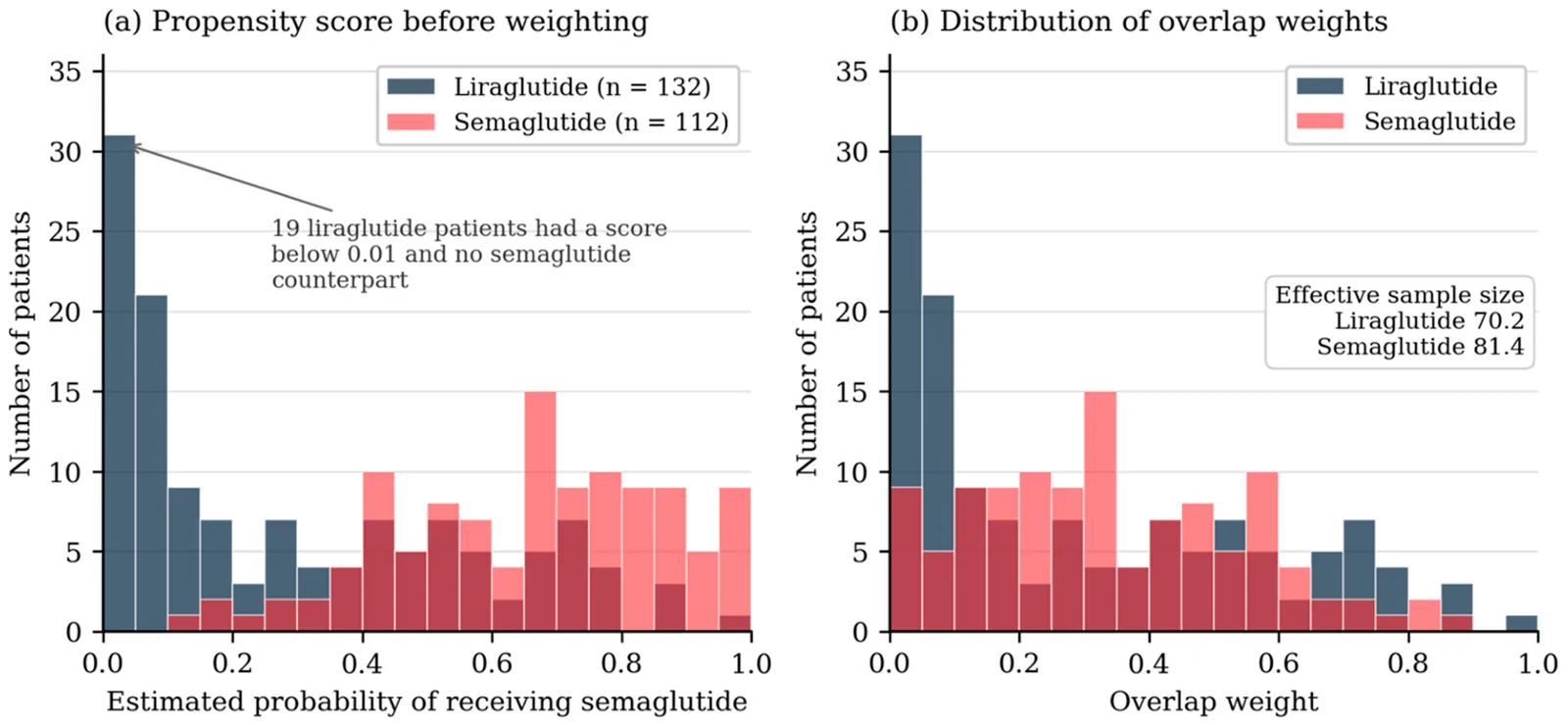
Distribution of the propensity score before weighting (**a**) and of the overlap weights (**b**), by treatment group. The dark pink shading represents the visual overlap between liraglutide and semaglutide distributions.

As noted in the Methods, exact balance is an algebraic property of the weighting method and not in itself evidence that confounding has been removed, so the distribution of the propensity score and of the weights is shown directly (Figure 2). The two distributions overlap substantially across the middle of the range but separate at the lower end. The median propensity score was 0.180 (IQR 0.054 to 0.513) in the liraglutide group and 0.690 (IQR 0.517 to 0.834) in the semaglutide group. Nineteen liraglutide-treated patients (14.4%) had a score below 0.01 and one semaglutide-treated patient (0.9%) had a score above 0.99. The 19 patients at the lower extreme received essentially zero weight and do not contribute to the weighted comparison. Effective sample size after weighting was 70.2 in the liraglutide group and 81.4 in the semaglutide group, compared with 132 and 112 patients with a propensity score. The weighted analysis therefore carries the precision of roughly 152 patients rather than 244.

### 3.6. Primary Outcome: Change in BMI

BMI fell in both groups, and the reduction was larger with semaglutide (Table 5). In the weighted, covariate-adjusted model, semaglutide produced a greater reduction of 0.60 kg/m^2^ at 3 months (95% CI 0.07 to 1.12; *p* = 0.026) and 1.42 kg/m^2^ at 6 months (95% CI 0.24 to 2.60; *p* = 0.019). Expressed as a proportion of baseline BMI, the difference was 1.9% at 3 months (95% CI 0.4 to 3.3; *p* = 0.012) and 4.9% at 6 months (95% CI 1.7 to 8.0; *p* = 0.003).

The mixed model for repeated measures, which used every recorded visit from 245 patients rather than only those with complete data, gave the same conclusion. The interaction between treatment group and visit was significant (*p* < 0.001). Estimated reductions in BMI were 0.51 kg/m^2^ at 3 months and 0.87 kg/m^2^ at 6 months with liraglutide, compared with 1.47 kg/m^2^ and 2.25 kg/m^2^ with semaglutide.

The finding was stable across specifications. Inverse probability weighting gave differences of 0.64 kg/m^2^ at 3 months (*p* = 0.013) and 1.73 kg/m^2^ at 6 months (*p* = 0.002). A generalised estimating equation with robust standard errors gave 0.65 kg/m^2^ at 3 months (*p* = 0.018). An unweighted model adjusted for the same covariates gave 0.77 kg/m^2^ at 3 months (*p* = 0.004), and 0.55 kg/m^2^ (*p* = 0.010) after excluding observations with a Cook distance above 4/*n*.

The observed difference of 1.42 kg/m^2^ at 6 months should be read against the precision available: the minimum difference the study could detect at that time point with 80% power was 1.30 kg/m^2^. The estimate is therefore close to the limit of what this sample can resolve, and the confidence interval deserves as much weight as the *p*-value.

### 3.7. Clinical Magnitude: Body Weight and Responder Analyses

Change in BMI is difficult to interpret at the bedside, so the same comparison is presented in kilograms, as percentage total body weight loss, and as the proportion of patients reaching recognised thresholds (Table 6).

Observed mean weight change at 6 months was −1.81 kg (SD 6.06) with liraglutide and −6.35 kg (SD 7.08) with semaglutide. In the weighted, covariate-adjusted model, the between-group difference was −1.70 kg at 3 months (95% CI −3.10 to −0.29; *p* = 0.018) and −3.66 kg at 6 months (95% CI −6.72 to −0.60; *p* = 0.020). As a percentage of baseline weight, the adjusted differences were −1.73% (95% CI −3.12 to −0.33; *p* = 0.015) and −3.94% (95% CI −6.97 to −0.91; *p* = 0.011).

The responder analyses show the same pattern more directly. At 3 months, 35.9% of semaglutide-treated patients had lost at least 5% of body weight compared with 12.4% of those receiving liraglutide (*p* < 0.001), and at 6 months the proportions were 56.5% and 24.7% (*p* < 0.001). For at least 10% loss, the proportions were 8.7% versus 1.1% at 3 months (*p* = 0.035) and 30.4% versus 4.9% at 6 months (*p* < 0.001). After overlap weighting and covariate adjustment, the odds ratios remained in the same direction but were less precisely estimated, reaching conventional significance for 5% loss at 3 months (adjusted odds ratio 2.86, 95% CI 1.13 to 7.25) and for 10% loss at 6 months (9.20, 95% CI 1.76 to 47.95) but not at the other two time points. With between 9 and 46 events, these weighted models are imprecise, and the unweighted proportions carry more information than the adjusted odds ratios.

The mixed model for body weight agreed with the weighted analysis (group-by-visit interaction *p* < 0.001). Estimated weight loss was 1.31 kg at 3 months and 2.37 kg at 6 months with liraglutide, against 3.90 kg and 5.91 kg with semaglutide.

### 3.8. Glycaemic Control

Only 18 semaglutide-treated patients contributed a 6-month HbA1c. No statistically detectable difference was observed, but this analysis is inconclusive and does not establish that the two agents have comparable glycaemic efficacy (Table 7).

### 3.9. Other Cardiometabolic Outcomes and Renal Function

Between-group differences in the other cardiometabolic outcomes at 3 months are shown in Table 8, with both raw and multiplicity-adjusted *p*-values. Only total cholesterol remained significant after correction for the false discovery rate, with semaglutide associated with a reduction of 0.71 mmol/L relative to liraglutide (adjusted *p* = 0.024). The apparent advantages in systolic blood pressure and triglycerides did not survive correction, and the mixed models for blood pressure, heart rate, and eGFR showed no significant interaction between group and visit.

Renal function did not change appreciably in either group. This should not be read as evidence of renal safety. Only 11 semaglutide-treated patients had an eGFR recorded at 6 months, and the laboratory reports all values above 90 mL/min/1.73 m^2^ as 91, so the distribution is censored at the top and cannot detect change within the normal range.

### 3.10. Documented Adverse Events

Adverse events were infrequent in both groups and did not differ significantly (Table 9). At least one adverse event was documented in 12.6% of liraglutide-treated and 8.2% of semaglutide-treated patients (*p* = 0.251). Gastrointestinal events were the most common in both groups, and no injection-site reaction was recorded in either.

Hypoglycaemia occurred in seven liraglutide-treated patients and one semaglutide-treated patient. The exact test *p*-value was 0.069, and after overlap weighting and adjustment for background insulin or sulfonylurea use, the difference was not significant (*p* = 0.345). Among liraglutide-treated patients, hypoglycaemia occurred in 2 of 49 without background insulin or sulfonylurea and 5 of 65 with it; the single semaglutide case was in a patient taking a sulfonylurea. With eight events in total, this comparison is exploratory and cannot support a conclusion about the intrinsic hypoglycaemia risk of either drug.

These rates are far lower than those reported when gastrointestinal symptoms are actively solicited in prospective trials, where they exceed 80% for both agents [15]. Adverse events here were captured passively from routine consultation notes, and liraglutide-treated patients had both longer exposure and more visits, which gave more opportunity for an event to be recorded. Similar crude proportions therefore cannot establish comparable safety.

### 3.11. Treatment Persistence and Discontinuation

Crude discontinuation was much higher with liraglutide: 66 of 135 patients (48.9%) stopped treatment, compared with 11 of 122 (9.0%) receiving semaglutide. That comparison is misleading, because median exposure was 17 months in the liraglutide group and 6 months in the semaglutide group, which gave liraglutide-treated patients almost three times as long in which discontinuation could occur (Table 10).

The unadjusted hazard ratio for discontinuation with semaglutide relative to liraglutide was 1.54 (95% CI 0.64 to 3.72; *p* = 0.333). In the primary model, adjusted for baseline covariates only, the hazard ratio was 1.60 (95% CI 0.61 to 4.19; *p* = 0.341), based on 244 patients and 76 events.

At a fixed horizon of 6 months, at which both groups had comparable observation time, 7 of 135 liraglutide-treated patients (5.2%) and 11 of 122 semaglutide-treated patients (9.0%) had discontinued (log-rank *p* = 0.188). The direction of this comparison is the opposite of the crude one.

**Figure 3:**
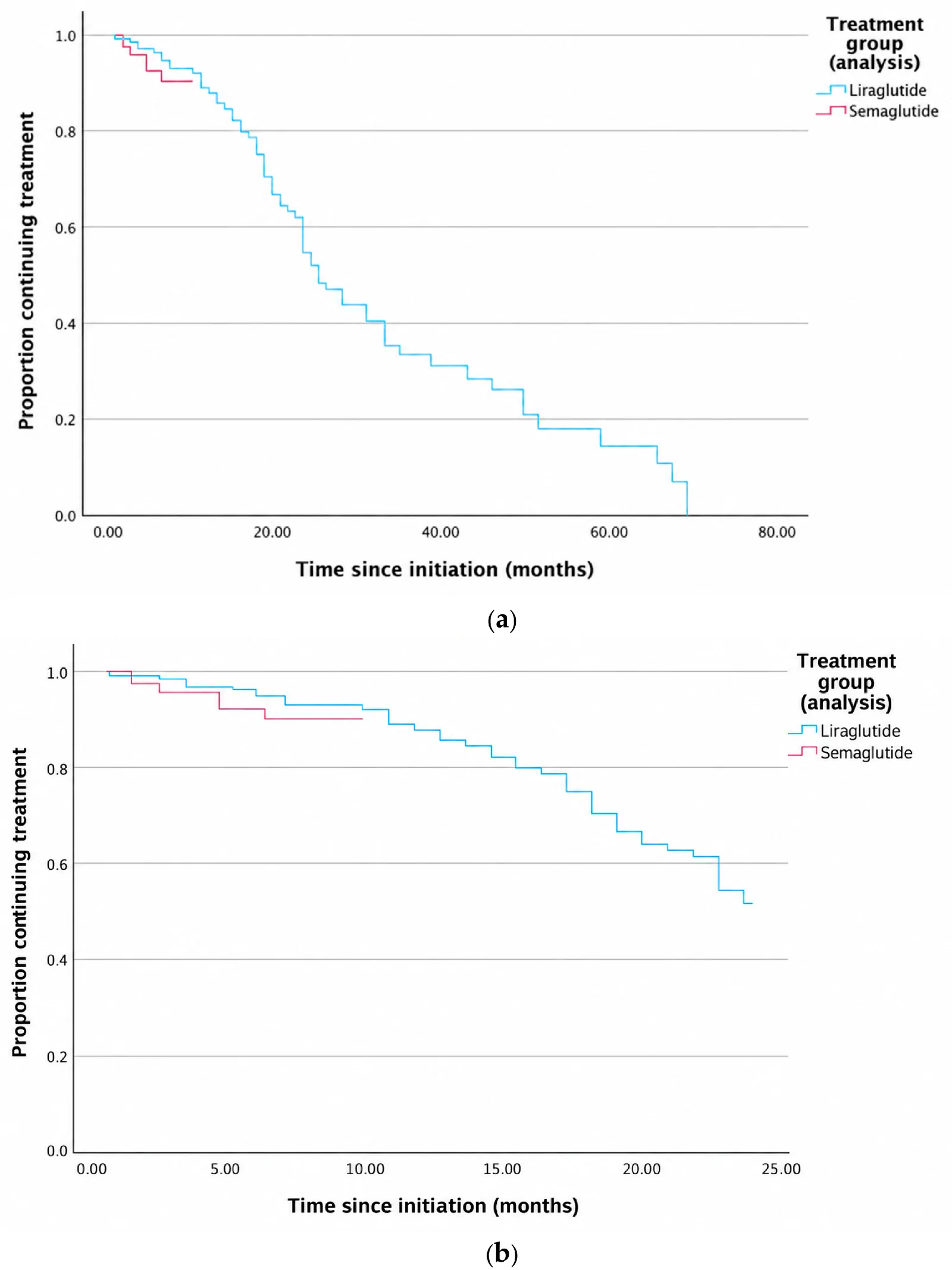
(**a**,**b**) Kaplan–Meier estimates of treatment persistence by group (entire timeline vs. capped at 24 months).

The reasons for and timing of discontinuation did differ. Among patients who stopped liraglutide, 95.5% did so more than 10 weeks after initiation, and the recorded reason was most often something other than adverse events or lack of effect. Among those who stopped semaglutide, 70.0% did so within the first four weeks, and adverse events were the reason in 70.0%. Semaglutide discontinuation was therefore an early, tolerability-driven event, while liraglutide discontinuation accumulated slowly over a much longer period. Figure 3a,b presents two Kaplan–Meier curves illustrating treatment persistence over the entire follow-up period and at 24 months, respectively.

### 3.12. Modal-Dose Subgroup Analysis

An exploratory comparison was made between patients maintained on the modal dose in each group, liraglutide 1.8 mg daily and semaglutide 1.0 mg weekly (Supplementary Table S1). This is not dose matching in a pharmacological sense, and maintenance dose is a post-baseline characteristic shaped by tolerability, response and clinician judgement, so restricting on it can introduce selection bias rather than remove confounding. The results are consistent with the main analysis and are presented as descriptive only.

### 3.13. Post Hoc Sensitivity Analyses

The primary BMI result was examined under six sensitivity analyses (Table 11).

Departure from missing at random. In the delta-adjusted pattern-mixture analysis, the 3-month difference remained significant until semaglutide non-completers were assumed to have done worse than completers by 1.75 kg/m^2^ (*p* = 0.050), and lost significance at 2.00 kg/m^2^ (*p* = 0.078). At 6 months, the finding persisted to a penalty of 2.00 kg/m^2^ (*p* = 0.035) and was lost at 2.50 kg/m^2^ (*p* = 0.357). For a patient of average height in this cohort, these tipping points correspond to roughly 5 kg and 7 kg, which are substantial departures from the missing-at-random assumption. The primary result is therefore reasonably robust, though not entirely protected from informative missingness.

The 14 patients who were sequentially exposed to both treatments were excluded from the analysis. In the 241 remaining patients, the 6-month results were unchanged (BMI −1.41 kg/m^2^, 95% CI −2.61 to −0.21, *p* = 0.021; percentage weight loss −3.91%, 95% CI −6.99 to −0.83, *p* = 0.013) and the mixed-model interaction remained significant (*p* < 0.001). The 3-month BMI difference attenuated to −0.53 kg/m^2^ (95% CI −1.06 to 0.00) and was no longer statistically significant (*p* = 0.051). The point estimate is close to that in the full cohort, and the loss of significance is consistent with a modest effect estimated in a smaller sample, but the 3-month result is the less secure of the two. Persistence was unchanged (hazard ratio 1.44, 95% CI 0.54 to 3.82; log-rank *p* = 0.458).

Four further sensitivity analyses were applied to the primary outcome. Inverse probability weighting, trimmed at the first and ninety-ninth percentiles, gave an adjusted difference of −0.64 kg/m^2^ at 3 months (95% CI −1.15 to −0.14; *p* = 0.013) and −1.73 kg/m^2^ at 6 months (95% CI −2.84 to −0.63; *p* = 0.002). An unweighted model adjusted for the same covariates gave −0.77 kg/m^2^ at 3 months (95% CI −1.30 to −0.25; *p* = 0.004) in 181 patients. A generalised estimating equation with robust standard errors, fitted as a check on the design-based variance estimator used in the primary model, gave −0.65 kg/m^2^ at 3 months (95% CI −1.18 to −0.11; *p* = 0.018), which is close to the primary estimate of −0.60 kg/m^2^. Model diagnostics raised no concern: variance inflation factors ranged from 1.15 to 1.63, and the largest Cook distance was 0.135. Refitting after excluding the observations whose Cook distance exceeded 4/*n* left 161 patients and gave −0.55 kg/m^2^ (95% CI −0.97 to −0.13; *p* = 0.010), so no single patient drives the result. All four estimates lie in the same direction and within about 0.2 kg/m^2^ of the primary estimate.

### 3.14. Liraglutide Trajectory to 12 Months

Because only the liraglutide group had 12-month data, a within-group model was fitted for that arm alone. BMI continued to fall, from an estimated 36.8 kg/m^2^ at baseline to 35.6 kg/m^2^ at 12 months (overall *p* < 0.001). HbA1c fell from 7.99% at baseline to 7.27% at 3 months (*p* < 0.001) but had returned to 7.72% by 12 months, and the difference from baseline at that point was no longer significant (*p* = 0.532). This is presented as a description of the liraglutide group and not as a comparison with semaglutide, for which no 12-month data exist.

## 4. Discussion

This retrospective cohort study compared semaglutide and liraglutide in Omani adults with overweight or obesity, with and without T2DM. After accounting for the substantial differences between the two groups in why treatment was started and how long it continued, semaglutide was associated with a modestly greater reduction in BMI at both 3 and 6 months. Glycaemic control did not differ. Of eight secondary cardiometabolic outcomes, only total cholesterol remained significant after correction for multiple testing. Adverse events were infrequent and similar in both groups, and the difference in hypoglycaemia lost significance once background glucose-lowering therapy was taken into account. The large apparent difference in discontinuation disappeared once the unequal length of follow-up was allowed for.

### 4.1. Effect on Body Weight

Weight reduction remains a principal therapeutic objective in obesity and T2DM, because sustained weight loss improves glycaemic control, cardiovascular risk factors and overall metabolic health [29,30]. In this cohort, semaglutide produced a greater reduction in BMI than liraglutide, and the estimate was stable across weighting schemes, covariate adjustment, a mixed model that used all available visits, and exclusion of influential observations.

The direction agrees with the randomised evidence, but the size of the difference does not. STEP 8 reported a between-group difference of 9.4 percentage points in body weight at 68 weeks [15]. The difference here was 1.9% of baseline BMI at 3 months and 4.9% at 6 months. Several things account for the gap. Follow-up was much shorter. Dose escalation was incomplete, and no patient reached semaglutide 2.4 mg. Treatment in routine care is not accompanied by the structured lifestyle support built into trial protocols. Real-world cohorts elsewhere have reported the same pattern of attenuated effect, and titration to maintenance doses is often incomplete outside trial settings [2,11,20,31]. The absolute effect seen here should therefore be read as what these drugs achieve in ordinary clinical practice in this setting, rather than as a test of their maximal efficacy.

Expressing the comparison in clinically meaningful units facilitates interpretation of the magnitude of the observed difference. The adjusted difference of 3.66 kg at 6 months, and the finding that 56.5% of semaglutide-treated patients reached 5% weight loss against 24.7% on liraglutide, place the difference above the threshold at which improvement in cardiovascular risk factors becomes measurable [22]. At the same time, the 3-month difference of 0.60 kg/m^2^ sits close to the smallest difference this study could detect and did not remain significant when the 14 switchers were excluded. Statistical significance and clinical importance are not the same thing, and the 6-month findings are the more secure of the two.

In this study, the modal doses were 1.0 mg weekly and 1.8 mg daily, and no patient received semaglutide 2.4 mg because that dose was unavailable in Oman. We would suggest, as a hypothesis rather than a conclusion, that the advantage seen in randomised trials may be attenuated under routine-care conditions. This cohort cannot test that hypothesis, because it differs from the trials in dose, indication, follow-up, patient selection and ascertainment at the same time.

### 4.2. Glycaemic Control

HbA1c improved in both groups, and no statistically significant difference between them was demonstrated at either time point, whether analysed as change from baseline in a weighted model or as a group-by-visit interaction in a mixed model. The same was true when the analysis was restricted to patients with diabetes.

Importantly, only 15 semaglutide-treated patients were treated primarily for diabetes, and 6-month HbA1c was available for just 18 patients in that group. A study with this much missing data and this few patients cannot distinguish absence of an effect from absence of information, so the glycaemic comparison should be read as inconclusive rather than as showing comparable efficacy.

Randomised evidence does favour semaglutide for glycaemic control. STEP 8 found larger reductions in HbA1c, fasting plasma glucose and insulin resistance with semaglutide than liraglutide [15], and the SUSTAIN programme reported greater HbA1c reductions than several active comparators [32]. Two features of this cohort make it poorly placed to detect such a difference. Semaglutide was prescribed almost entirely for weight management and at the 1.0 mg dose licenced for T2DM, rather than the 2.4 mg dose used for chronic weight management [33]. Only 15 semaglutide-treated patients were treated primarily for diabetes, and 6-month HbA1c was available for just 18 patients in that group. A null result under these conditions is uninformative about the comparative glycaemic efficacy of the two agents.

One observation in the liraglutide group is worth recording. HbA1c fell by 3 months, then drifted back towards baseline by 12 months, with no significant difference from baseline at that point. Whether this reflects waning effect, changes in background therapy, or the selection of patients who remained in follow-up cannot be determined from these data, but it does suggest that early glycaemic response should not be assumed to persist.

### 4.3. Documented Adverse Events and Tolerability

Gastrointestinal symptoms were the most frequently recorded adverse events in both groups, with no significant difference between them. That is consistent with trials and meta-analyses showing comparable gastrointestinal tolerability between the two agents [11,15,34], and with pharmacovigilance analyses of the FAERS database in which gastrointestinal symptoms predominate for both [35,36].

The overall event rate here, around 8% to 13%, is an order of magnitude below the 80% and above reported when symptoms are systematically solicited in trials [15]. This reflects the method of capture rather than better tolerance. Events were recorded only if a clinician documented them during routine care. Liraglutide-treated patients attended a median of three visits over 17 months, while semaglutide-treated patients attended a median of one visit over 6 months, so the two groups did not have equal opportunity for an event to be documented. Similar crude proportions cannot be taken as evidence of comparable safety, and this study is not able to make a safety comparison between the two drugs.

Hypoglycaemia was recorded in 7 liraglutide-treated patients and 1 semaglutide-treated patient. The unadjusted exact test gave *p* = 0.069, and once background insulin or sulfonylurea use was taken into account, the difference was not significant. Five of the seven liraglutide cases and the single semaglutide case were in patients receiving insulin or a sulfonylurea. GLP-1 RAs stimulate insulin secretion in a glucose-dependent manner and carry a low intrinsic risk of hypoglycaemia, so the most plausible explanation is the distribution of background therapy rather than a difference between the agents [37,38]. With eight events in total, this finding is exploratory. Its practical message is the familiar one, that background glucose-lowering therapy should be reviewed when a GLP-1 RA is started.

### 4.4. Adherence and Persistence

Adherence and persistence are distinct constructs and behaved differently in this cohort [23]. Documented adherence, meaning a clinician record that the patient was taking the drug as prescribed, was higher in the semaglutide group (95.9% versus 83.7%). Persistence, meaning the time from initiation to discontinuation, did not differ once the unequal length of follow-up was taken into account.

In the current study, this is described consistently as documented adherence because that is what was measured: a binary clinician judgement recorded in the consultation note, not a refill-based metric such as proportion of days covered or medication possession ratio. The difference may reflect the weekly dosing interval, but it may equally reflect differences in follow-up intensity, documentation practice, indication and exposure, and this study cannot separate those explanations. It should not be read as establishing intrinsically superior adherence with semaglutide.

After accounting for differences in time at risk, the observed difference was no longer statistically significant. However, this finding should not be interpreted as evidence that unequal follow-up fully accounts for the initial difference. Rather, the present cohort provides no evidence of a difference in treatment persistence, while its ability to detect such a difference was limited.

Wider evidence still favours weekly dosing for persistence. Polonsky and colleagues found higher persistence with once-weekly than once-daily GLP-1 RAs at 6 and 12 months [16,17], a meta-analysis reached the same conclusion [18], and a large claims analysis reported one-year persistence of 47.1% for semaglutide against 19.2% for liraglutide [19]. Those studies followed patients for at least a year with a consistent definition of a treatment gap. The present cohort could not reproduce that design, because semaglutide had been available for a median of only 6 months. Our findings should be read as showing that this cohort was not able to detect a persistence difference, rather than as evidence against one.

The pattern of discontinuation is more informative than its overall frequency. Seven of the ten semaglutide discontinuations with documented timing occurred within four weeks and were attributed to adverse events, while liraglutide discontinuations accumulated slowly and were mostly attributed to other reasons. Early tolerability-driven dropout is well described for GLP-1 RAs and is a recognised target for closer support during initiation and titration [39,40].

### 4.5. Other Cardiometabolic Parameters and Renal Function

Total cholesterol was the only secondary outcome for which a between-group difference survived correction for multiple testing, with semaglutide associated with a reduction of 0.71 mmol/L at 3 months relative to liraglutide. The mixed model showed the same pattern, with total cholesterol in the semaglutide group falling at 3 months and returning towards baseline by 6 months, a trajectory based on only 12 semaglutide-treated patients at the later visit and therefore fragile.

Renal function appeared stable in both groups, but this study cannot support a statement about renal safety. Only 11 semaglutide-treated patients had an eGFR at 6 months, and the laboratory censors all values above 90 mL/min/1.73 m^2^ at 91, so most of the cohort sits at the ceiling of the reported range and change within the normal range is undetectable. The favourable renal profile of semaglutide reported in PIONEER and SUSTAIN [41,42] is not confirmed or contradicted by these data.

### 4.6. Generalisability

Almost 90% of semaglutide prescriptions in this cohort were for weight management, yet no patient reached the 2.4 mg weekly dose licenced for that indication, because it was not available in Oman during the study period. Patients were treated with the 1.0 mg dose licenced for T2DM. The comparison presented here is therefore between liraglutide at its usual maintenance dose and semaglutide at a dose below the one intended for weight management, and it is likely to understate what semaglutide can achieve when the higher dose is available. Findings should be applied only to settings with a similar formulary.

### 4.7. Limitations

This study has several limitations beyond those inherent to retrospective design. Confounding by indication is the most important. Semaglutide was prescribed for weight management in 87.7% of patients against 55.6% for liraglutide, and although overlap weighting balanced every measured covariate exactly, weighting cannot address confounders that were not recorded, such as socioeconomic status, dietary intervention, physical activity, or the clinical reasoning behind each prescribing decision. Also, positivity is a related limitation. Nineteen liraglutide-treated patients had no semaglutide counterpart and contributed nothing to the weighted comparison, and the effective sample size after weighting was 70.2 and 81.4 in the two groups. The weighted estimate applies to the subset of patients for whom either drug could plausibly have been chosen, not to the cohort as a whole. Additionally, exposure differed markedly, with a median of 17 months for liraglutide and 6 months for semaglutide, and follow-up intensity differed with it. This affects every comparison in different ways: it inflates crude discontinuation in the liraglutide group, it gives that group more opportunity for adverse events to be documented, and it means the 6-month effectiveness comparison is made between a liraglutide group that had already been on treatment for some time and a semaglutide group closer to initiation. Missing follow-up data were substantial and were related to observed baseline characteristics, so they are unlikely to be missing completely at random. The mixed model addresses this under a missing-at-random assumption, but that assumption cannot be verified, and missingness may depend on the unobserved values themselves. Semaglutide had no 12-month data at all, so no comparison beyond 6 months is possible. The pattern-mixture analysis shows the primary result holds unless non-completers in the semaglutide group did substantially worse than completers, but it cannot rule out a larger departure. Adverse events and adherence were captured passively from routine records rather than by systematic solicitation, which almost certainly understates both and may do so unequally between groups. Adherence was a binary clinician judgement rather than a calculated measure such as proportion of days covered, so it is not directly comparable with claims-based studies. Hypoglycaemia rested on eight events in total and is exploratory. Background glucose-lowering therapy was not documented by drug name for 21 liraglutide-treated patients, so the reported distribution of insulin and sulfonylurea use is incomplete, and those patients fell outside the region of overlap and did not contribute to the weighted comparison. The study was adequately powered only for the primary endpoint; the secondary, glycaemic and adverse-event comparisons are underpowered and should be read as hypothesis-generating. The responder analyses at the 10% threshold rest on 9 and 18 events and their weighted odds ratios are correspondingly imprecise. The modal-dose subgroup analysis was not prespecified and compares doses that are not pharmacologically equivalent, with maintenance dose being a post-baseline characteristic shaped by tolerability and response. It is descriptive only. Finally, this is a single-centre tertiary hospital cohort of 257 patients, with no patient on semaglutide 2.4 mg, and the comparisons are associative.

## 5. Conclusions

In this real-world Omani cohort, semaglutide was associated with a modestly greater reduction in BMI and body weight than liraglutide at 3 and 6 months, and with a higher proportion of patients reaching clinically meaningful weight loss thresholds. The difference persisted across several analytical approaches and remained robust to moderate departures from the missing-at-random assumption, although the 3-month result was sensitive to exclusion of the patients who had received both drugs. The comparison of glycaemic control was inconclusive rather than negative, and of eight secondary cardiometabolic outcomes only total cholesterol remained significant after correction for multiple testing. Documented adverse events were infrequent and similar in both groups, although passive ascertainment and unequal surveillance mean this study cannot establish comparable safety. Documented adherence was higher with semaglutide, while no difference in persistence was statistically apparent once time at risk was taken into account.

These findings are associative and come from a single centre where the 2.4 mg weekly dose of semaglutide was not available, so the two drugs were not compared at pharmacologically equivalent doses. They add regional data from an under-represented population and describe what these agents achieved as they were actually prescribed. Whether the advantages seen in randomised trials are attenuated in routine practice is a hypothesis these data raise rather than one they can test. Prospective studies with equal follow-up, systematic adverse-event ascertainment, refill-based adherence measurement and access to obesity-equivalent doses are needed.

## Figures and Tables

**Table 1:** Baseline characteristics of the analysed cohort.

Characteristic	Liraglutide (*n* = 135)	Semaglutide (*n* = 122)	*p*
Age, years, M (SD)	46.9 (14.1)	50.2 (14.4)	0.055
Female, *n* (%)	82 (60.7)	79 (64.8)	0.507
Weight, kg, Mdn [IQR]	92.6 [81.0–110.0]	97.4 [82.0–113.0]	0.367
BMI, kg/m^2^, Mdn [IQR] ^a^	35.4 [31.0–41.6]	36.2 [31.8–41.8]	0.298
BMI category, *n* (%) ^a^			0.546
<25 kg/m^2^	6 (4.5)	2 (1.8)	
25.0–29.9	18 (13.6)	15 (13.4)	
30.0–34.9	38 (28.8)	32 (28.6)	
35.0–39.9	33 (25.0)	23 (20.5)	
≥40.0	37 (28.0)	40 (35.7)	
HbA1c, %, Mdn [IQR] ^a^	7.9 [6.0–9.4]	7.8 [6.2–9.3]	0.701
Systolic BP, mmHg, Mdn [IQR] ^a^	129.5 [119–143]	130.0 [118–143]	0.787
Diastolic BP, mmHg, Mdn [IQR] ^a^	70 [63–78]	71 [64–82]	0.590
Heart rate, bpm, Mdn [IQR] ^a^	82 [76–92]	82 [75–92]	0.911
Triglycerides, mmol/L, Mdn [IQR] ^a^	1.45 [1.00–2.00]	1.40 [1.10–2.00]	0.544
Total cholesterol, mmol/L, Mdn [IQR] ^a^	4.35 [3.50–5.00]	4.70 [3.90–5.80]	0.008
eGFR, mL/min/1.73 m^2^, Mdn [IQR] ^ab^	91.0 [82.0–91.0]	90.0 [90.0–91.0]	0.110
Type 2 diabetes, *n* (%)	100 (74.1)	88 (72.1)	0.726
Hypertension, *n* (%)	61 (45.2)	73 (59.8)	0.019
Dyslipidaemia, *n* (%)	65 (48.1)	81 (66.4)	0.003
Cardiovascular disease, *n* (%)	33 (24.4)	16 (13.1)	0.021
NAFLD, *n* (%)	9 (6.7)	25 (20.5)	0.001
Hypothyroidism, *n* (%)	22 (16.3)	27 (22.1)	0.234
Previous bariatric surgery, *n* (%)	11 (8.1)	11 (9.0)	0.804

BMI, body mass index; BP, blood pressure; eGFR, estimated glomerular filtration rate; IQR, interquartile range; M, mean; Mdn, median; NAFLD, non-alcoholic fatty liver disease; SD, standard deviation. Continuous variables were compared with the Mann–Whitney U test; categorical variables with the Pearson chi-square test. ^a^ Denominators vary because of missing values: BMI 132 and 112; HbA1c 120 and 119; blood pressure 130 and 119; heart rate 130 and 120; triglycerides 106 and 109; total cholesterol 106 and 111; eGFR 123 and 119. ^b^ The laboratory reports eGFR values above 90 as 91, so the upper part of this distribution is censored.

**Table 2:** Treatment characteristics and concomitant glucose-lowering therapy.

Characteristic	Liraglutide (*n* = 135)	Semaglutide (*n* = 122)	*p*
Indication, *n* (%)			<0.001
Weight management	75 (55.6)	107 (87.7)	
Type 2 diabetes	57 (42.2)	15 (12.3)	
Other	3 (2.2)	0 (0.0)	
Treatment duration, months, Mdn [IQR]	17 [8–28]	6 [4–7]	<0.001
Follow-up visits, *n*, Mdn [IQR]	3 [2–6]	1 [1–1]	<0.001
Documented adherence, *n* (%)	113 (83.7)	117 (95.9)	0.001
Concomitant therapy documentation, *n* (%)			<0.001
Drug names recorded	87 (64.4)	90 (73.8)	
No concomitant therapy recorded	27 (20.0)	32 (26.2)	
Therapy present, names not recorded	21 (15.6)	0 (0.0)	
Insulin, *n* (%) ^a^	52 (45.6)	42 (34.4)	0.079
Sulfonylurea, *n* (%) ^a^	28 (24.6)	40 (32.8)	0.163
Metformin, *n* (%) ^a^	67 (58.8)	77 (63.1)	0.494
SGLT2 inhibitor, *n* (%) ^a^	49 (43.0)	67 (54.9)	0.067
DPP-4 inhibitor, *n* (%) ^a^	1 (0.9)	3 (2.5)	0.623

DPP-4, dipeptidyl peptidase-4; IQR, interquartile range; Mdn, median; SGLT2, sodium-glucose co-transporter-2. ^a^ Denominators are 114 for liraglutide and 122 for semaglutide, because background therapy was recorded without drug names for 21 liraglutide-treated patients. The DPP-4 comparison used the Fisher exact test.

**Table 3:** Number of patients contributing data at each visit.

Outcome	Baseline L/S	3 Months L/S	6 Months L/S	12 Months L/S
BMI	132/112	89/93	81/46	87/0
HbA1c	120/119	75/57	72/18	83/0
Systolic blood pressure	130/119	100/86	99/29	96/0
Diastolic blood pressure	130/119	101/88	98/27	96/0
Triglycerides	106/109	55/44	50/14	69/0
Total cholesterol	106/111	55/43	49/12	67/0
eGFR	123/119	82/51	90/11	90/0
Heart rate	130/120	100/87	99/26	96/0

L, liraglutide (*n* = 135); S, semaglutide (*n* = 122); BMI, body mass index; eGFR, estimated glomerular filtration rate. Semaglutide was introduced later in the study period, so no patient in that group reached a 12-month assessment.

**Table 4:** Standardised mean differences before and after overlap weighting.

Covariate	Before Weighting	After Weighting
Age	0.198	0.000
Male sex	−0.039	0.000
Baseline BMI	0.208	0.000
Type 2 diabetes	−0.063	0.000
Indication weight management	0.788	0.000
Hypertension	0.268	0.000
Dyslipidaemia	0.325	0.000
Cardiovascular disease	−0.306	0.000
NAFLD	0.427	0.000
Insulin or sulfonylurea use	−0.169	0.000

BMI, body mass index; NAFLD, non-alcoholic fatty liver disease. A positive value indicates a higher mean in the semaglutide group. Values are calculated in the 244 patients with a complete covariate set. An absolute value below 0.10 is conventionally taken to indicate balance.

**Table 5:** Change in BMI from baseline by treatment group.

Analysis	Liraglutide, kg/m^2^ (95% CI)	Semaglutide, kg/m^2^ (95% CI)	Difference (95% CI); *p*
Weighted, covariate-adjusted model (primary)			
3 months	−0.99 (−1.46, −0.53)	−1.59 (−2.22, −0.96)	−0.60 (−1.12, −0.07); 0.026
6 months	−1.39 (−2.32, −0.46)	−2.81 (−4.30, −1.32)	−1.42 (−2.60, −0.24); 0.019
Percentage change from baseline BMI			
3 months	−1.84% (−2.85, −0.82)	−3.70% (−4.74, −2.67)	−1.87% (−3.32, −0.42); 0.012
6 months	−0.98% (−2.46, 0.50)	−5.83% (−8.65, −3.01)	−4.85% (−8.04, −1.66); 0.003
Mixed model for repeated measures (*n* = 245 patients)			
3 months	−0.51 (−0.89, −0.13)	−1.47 (−1.86, −1.09)	Interaction *p* < 0.001
6 months	−0.87 (−1.52, −0.22)	−2.25 (−3.02, −1.48)	
Sensitivity analyses, difference at 3 and 6 months			
Inverse probability weighting	—	—	−0.64 (*p* = 0.013); −1.73 (*p* = 0.002)
Unweighted, covariate-adjusted	—	—	−0.77 (*p* = 0.004)
Excluding influential observations	—	—	−0.55 (*p* = 0.010)

BMI, body mass index; CI, confidence interval. A negative value denotes a reduction. The difference is semaglutide minus liraglutide, so a negative difference favours semaglutide. The primary model uses overlap weights and adjusts for age, sex, baseline BMI, diabetes, indication, hypertension, dyslipidaemia, cardiovascular disease and NAFLD. Estimates are based on 181 patients at 3 months and 127 at 6 months.

**Table 6:** Clinical magnitude of weight change and responder analyses.

Outcome	Liraglutide	Semaglutide	Difference or OR (95% CI); *p*
Observed change in body weight, M (SD)			
3 months, kg	−1.32 (3.41), *n* = 89	−3.87 (4.77), *n* = 92	—
6 months, kg	−1.81 (6.06), *n* = 81	−6.35 (7.08), *n* = 46	—
Weighted, covariate-adjusted estimates			
Weight, 3 months, kg	−2.58 (−3.82, −1.35)	−4.28 (−6.04, −2.53)	−1.70 (−3.10, −0.29); 0.018
Weight, 6 months, kg	−3.56 (−5.83, −1.29)	−7.22 (−10.83, −3.60)	−3.66 (−6.72, −0.60); 0.020
Weight loss, 3 months, %	−2.43 (−3.71, −1.16)	−4.16 (−5.83, −2.49)	−1.73 (−3.12, −0.33); 0.015
Weight loss, 6 months, %	−3.65 (−5.94, −1.36)	−7.59 (−11.26, −3.92)	−3.94 (−6.97, −0.91); 0.011
Responder analyses, *n* (%)			
≥5% loss, 3 months	11/89 (12.4)	33/92 (35.9)	2.86 (1.13, 7.25); <0.001
≥10% loss, 3 months	1/89 (1.1)	8/92 (8.7)	2.94 (0.32, 26.89); 0.035
≥5% loss, 6 months	20/81 (24.7)	26/46 (56.5)	2.37 (0.85, 6.57); <0.001
≥10% loss, 6 months	4/81 (4.9)	14/46 (30.4)	9.20 (1.76, 47.95); <0.001

CI, confidence interval; M, mean; OR, odds ratio; SD, standard deviation. Negative values denote a reduction; the difference is semaglutide minus liraglutide. Weighted estimates use overlap weights with adjustment for baseline BMI, age, sex, diabetes, indication, hypertension, dyslipidaemia, cardiovascular disease, and non-alcoholic fatty liver disease. For the responder rows, the odds ratio is from the weighted adjusted model and the *p*-value is the Fisher exact test on the unweighted proportions. The two models for 10% loss were reduced to treatment group and baseline BMI because only 9 and 18 events were available.

**Table 7:** Change in HbA1c from baseline by treatment group.

Time Point	Liraglutide, % (95% CI)	Semaglutide, % (95% CI)	Difference (95% CI); *p*
3 months	−0.67 (−1.02, −0.32)	−1.03 (−1.35, −0.71)	−0.36 (−0.83, 0.11); 0.132
6 months	−0.73 (−1.13, −0.33)	−1.28 (−1.91, −0.64)	−0.55 (−1.32, 0.22); 0.158

CI, confidence interval. Weighted, baseline-adjusted estimates from the Complex Samples general linear model. Based on 128 patients at 3 months and 82 at 6 months. The corresponding mixed model gave a group-by-visit interaction *p* = 0.360 in the whole cohort and *p* = 0.236 among patients with diabetes.

**Table 8:** Between-group differences in secondary cardiometabolic outcomes at 3 months.

Outcome	Difference (95% CI) ^a^	Raw *p*	Adjusted *p* ^b^
Total cholesterol, mmol/L	−0.71 (−1.17, −0.25)	0.003	0.024
Systolic blood pressure, mmHg	−5.70 (−12.03, 0.62)	0.077	0.229
Triglycerides, mmol/L	−0.76 (−1.63, 0.11)	0.086	0.229
HbA1c at 3 months, %	−0.36 (−0.83, 0.11)	0.132	0.253
HbA1c at 6 months, %	−0.55 (−1.32, 0.22)	0.158	0.253
Heart rate, bpm	−1.51 (−4.97, 1.95)	0.392	0.523
Diastolic blood pressure, mmHg	0.97 (−2.76, 4.70)	0.609	0.696
eGFR, mL/min/1.73 m^2^	0.30 (−2.60, 3.19)	0.840	0.840

CI, confidence interval; eGFR, estimated glomerular filtration rate. ^a^ Semaglutide minus liraglutide, from weighted models adjusted for the baseline value of the outcome; a negative value favours semaglutide. ^b^ Benjamini–Hochberg false discovery rate correction across these eight secondary outcomes. The primary BMI outcome was prespecified and is not included in the correction.

**Table 9:** Adverse events and adherence.

Outcome	Liraglutide (*n* = 135)	Semaglutide (*n* = 122)	*p*
Any adverse event, *n* (%)	17 (12.6)	10 (8.2)	0.251
Nausea	9 (6.7)	6 (4.9)	0.550
Vomiting	4 (3.0)	2 (1.6)	0.686
Diarrhoea	4 (3.0)	0 (0.0)	0.124
Constipation	3 (2.2)	1 (0.8)	0.624
Injection-site reaction	0 (0.0)	0 (0.0)	—
Hypoglycaemia, *n* (%)	7 (5.2)	1 (0.8)	0.069
With insulin or sulfonylurea	5/65 (7.7)	1/62 (1.6)	—
Without insulin or sulfonylurea	2/49 (4.1)	0/60 (0.0)	—
Documented adherence, *n* (%)	113 (83.7)	117 (95.9)	0.001

Vomiting, diarrhoea, constipation and hypoglycaemia were compared with the Fisher exact test because of low expected cell counts. After overlap weighting and adjustment for background insulin or sulfonylurea use, the between-group difference in hypoglycaemia was not significant (*p* = 0.345). Background therapy was not documented by name for 21 liraglutide-treated patients, so the subgroup denominators sum to 114 rather than 135.

**Table 10:** Treatment persistence and reasons for discontinuation.

Measure	Liraglutide	Semaglutide	*p*
Discontinued at any time, *n* (%)	66/135 (48.9)	11/122 (9.0)	— ^a^
Median exposure, months	17	6	<0.001
Median time to discontinuation, months	26.0 (22.5–29.5)	Not reached	0.328 ^b^
Unadjusted HR (semaglutide vs. liraglutide)	1.00 (reference)	1.54 (0.64–3.72)	0.333
Adjusted HR ^c^	1.00 (reference)	1.63 (0.62–4.29)	0.326
Discontinued within 6 months, *n* (%)	7 (5.2)	11 (9.0)	0.188 ^b^
Timing of discontinuation, *n* (%) ^d^			<0.001
1–4 weeks	2 (3.0)	7 (70.0)	
5–10 weeks	1 (1.5)	2 (20.0)	
>10 weeks	63 (95.5)	1 (10.0)	
Reason for discontinuation, *n* (%) ^d^			<0.001
Adverse events	9 (13.6)	7 (70.0)	
Lack of effect	7 (10.6)	0 (0.0)	
Cost	0 (0.0)	1 (10.0)	
Other	50 (75.8)	2 (20.0)	

HR, hazard ratio. ^a^ The crude proportions are reported for transparency but are not compared statistically, because exposure differed almost threefold. ^b^ Log-rank test. ^c^ Adjusted for age, sex, baseline BMI, diabetes, indication, background insulin or sulfonylurea use, and documented adherence (*n* = 244). ^d^ Denominators are the patients who discontinued (66 liraglutide, 10 semaglutide with a documented reason and timing); one semaglutide discontinuation had neither recorded.

**Table 11:** Sensitivity analyses for the primary outcome.

Analysis	BMI Change, 3 Months	BMI Change, 6 Months
Primary model	−0.60 (−1.12, −0.07); 0.026	−1.42 (−2.60, −0.24); 0.019
Delta-adjusted pattern mixture (departure from missing at random)		
delta = 0	−0.72 (−1.14, −0.30); <0.001	−1.80 (−2.31, −1.29); <0.001
delta = 1.00 kg/m^2^	−0.56 (−1.00, −0.13); 0.011	−1.18 (−1.69, −0.68); <0.001
delta = 1.75 kg/m^2^	−0.44 (−0.89, 0.00); 0.050	—
delta = 2.00 kg/m^2^	−0.40 (−0.85, 0.05); 0.078	−0.56 (−1.08, −0.04); 0.035
delta = 2.50 kg/m^2^	—	−0.25 (−0.79, 0.28); 0.357
Other sensitivity analyses		
Excluding the 14 switchers (*n* = 241)	−0.53 (−1.06, 0.00); 0.051	−1.41 (−2.61, −0.21); 0.021
Inverse probability weighting	−0.64; 0.013	−1.73; 0.002
Unweighted, covariate-adjusted	−0.77; 0.004	—
Excluding influential observations	−0.55; 0.010	—

Values are the adjusted between-group difference (95% CI); *p*. A negative value favours semaglutide. The pattern-mixture analysis replaces missing values with the observed group mean and adds delta to the semaglutide non-completers only; the tipping point is the value of delta at which the difference ceases to be significant. Em dashes indicate a value not applicable at that time point.

## Data Availability

The original contributions presented in this study are included in the article/Supplementary Material. Further inquiries can be directed to the corresponding authors.

## Supplementary Materials

The following supporting information can be downloaded at: https://www.mdpi.com/article/10.3390/jcm15207797/s1, Supplementary Table S1. Modal-dose subgroup: change in BMI from baseline.
