# Supplementary material for: Real-World Effectiveness, Documented Adverse Events, and Treatment Adherence of Semaglutide and Liraglutide in Omani Patients: A Retrospective Observational Study
Source: J Clin Med. 2026 Oct 9;15(20):7797. doi: 10.3390/jcm15207797 (PMC13705067; doi:10.3390/jcm15207797)
Supplement: Supplementary file 1 [file jcm-15-07797-s001.zip › jcm-4549116-supplementary.pdf]

Supplementary Table S1. Modal-dose subgroup: change in BMI from baseline.

Time point	Liraglutide 1.8 mg, M (SD)	Semaglutide 1.0 mg, M (SD)	n
3 months	−0.48 (1.18)	−1.47 (1.81)	60 / 81
6 months	−0.20 (1.56)	−2.32 (2.87)	56 / 41

M, mean; SD, standard deviation. Values are change in BMI in kg/m^2; a negative value denotes a reduction. This subgroup analysis was not prespecified and no formal between-group test is reported, because the subgroups are defined by an achieved maintenance dose rather than by randomisation.
